# Population Genetics of the Endemic Hawaiian Species *Chrysodracon hawaiiensis* and *Chrysodracon auwahiensis* (Asparagaceae): Insights from RAPD and ISSR Variation

**DOI:** 10.3390/ijms17081341

**Published:** 2016-08-16

**Authors:** Pei-Luen Lu, Mitsuko Yorkson, Clifford W. Morden

**Affiliations:** 1 Department of BioResources, Da-Yeh University, Changhua 51591, Taiwan; 2 Department of Botany, University of Hawaii at Mānoa, Honolulu, HI 96822, USA; mitsuko@hawaii.rr.com (M.Y.); cmorden@hawaii.edu (C.W.M.)

**Keywords:** *Chrysodracon*, conservation, Hawaiian species, ISSR, *Pleomele*, population genetics, RAPD

## Abstract

The genus *Chrysodracon* has six endemic species in the Hawaii Islands. *Chrysodracon hawaiiensis* is endemic to Hawaii Island and was described as a distinct species in 1980. It was listed as an endangered species on the International Union for the Conservation of Nature and Natural Resources (IUCN) Red List in 1997. This woody plant species was, at one time, common in exposed dry forests, but it became very rare due to grazing pressure and human development. The tree species *Chrysodracon auwahiensis* (*C. auwahiensis*), endemic to Maui and Molokai, still has large adult populations in dry lands of the islands, but unfortunately no regeneration from seed has been reported in those areas for many years. The two endemic species were examined using the molecular technique of random amplified polymorphic DNA (RAPD) and inter simple sequence repeats (ISSR) to determine the genetic structure of the populations and the amount of variation. Both species possess similar genetic structure. Larger and smaller populations of both species contain similar levels of genetic diversity as determined by the number of polymorphic loci, estimated heterozygosity, and Shannon’s index of genetic diversity. Although population diversity of *Chrysodracon hawaiiensis* (*C. hawaiiensis*) is thought to have remained near pre-disturbance levels, population size continues to decline as recruitment is either absent or does not keep pace with senescence of mature plants. Conservation recommendations for both species are suggested.

## 1. Introduction

The Hawaiian Islands include a high percentage of endemic species and are one of 25 biodiversity hotspots in the world [1,2,3,4]. Although many genera are species-rich in Hawaii and have special evolutionary histories, few of them have been studied in detail [4,5,6]. Many Pacific Island species, including those from the Hawaiian Islands, have a fragile existence. This is often due to their populations being scattered broadly within or across different islands and a limited genetic diversity due to their recent colonization, isolation from the source population, and/or the population size being restricted within island environments [4,5,7,8]. A consequence of this fragility has resulted in many endemic Hawaiian plant species having become endangered and the level of genetic diversity present becoming severely reduced compounding the problems for species recovery [9,10,11]. For example, the Hawaiian dry forests have been seriously reduced due to habitat loss from commercial or agricultural development and the spread of invasive plant and animal species [5,12]. Notably, more than 90% of Hawaiian dry forests are already lost [13] and 50% of the extant Hawaiian endemic flora is listed as endangered or rare in the International Union for the Conservation of Nature and Natural Resources (IUCN) [14] or by the US Fish and Wildlife Service (USFWS) [15]. Therefore, the study and conservation of genetic resources in populations, species, and ecosystems are essential to maintaining biodiversity and population dynamics.

**Figure 1:**
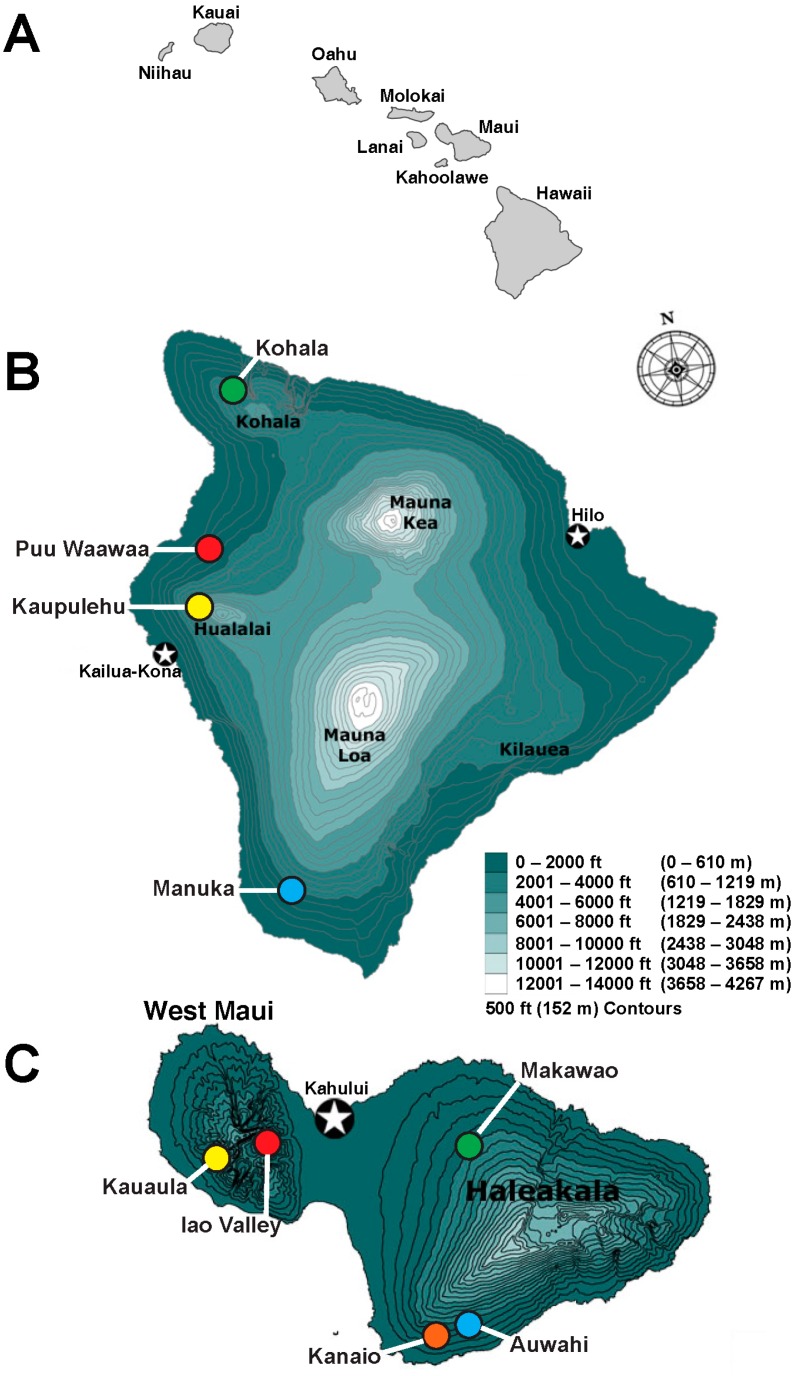
Locations of populations used in *Chrysodracon* genetic studies. (**A**) The eight Hawaiian Islands; (**B**) topographic map of Hawaii Island; (**C**) topographic map of Maui Island. Populations are demarcated with the color used in the principal coordinate analysis (PCO) analysis. Names of volcanoes on each island are indicated for Hawaii (5) and Maui (2); principle cities associated with each islands are indicated with a star (★).

The Hawaiian endemic genus *Chrysodracon* (Jankaski) Lu and Morden (Asparagaceae), species previously included among the widely distributed tropical genus *Pleomele* Salisbury, has six endemic species in the Hawaiian Islands [16,17]. *Chrysodracon hawaiiensis* (Degener and Degener) Lu and Morden was distinguished as a species in 1980 [18]. Unfortunately, populations of this species have declined rapidly in the past few decades and the USFWS listed *C. hawaiiensis* (as *Pleomele hawaiiensis*) as an endangered species in 1996 [15]. The IUCN also placed it on their red list of endangered and threatened species in 1997 [14]. *Chrysodracon hawaiiensis* exists in only 6–8 populations totaling approximately 300–400 individuals in sunny dry forests on the leeward side of Hawaii Island [15] (Figure 1). The largest extant wild population with approximately 200 individuals is located at Puu Waawaa [15]. This species has a unique ability to grow in young lava substrates often on steep slopes. To date, nothing is known about the genetic structure of this species. Due to its rarity and small population sizes, it may possibly go extinct or become more severely restricted in distribution within the next few decades if appropriate conservation management are not adopted.

Presently, *C. hawaiiensis* is the only species of *Chrysodracon* recognized as occurring on Hawaii Island [17]. However, St. John [19] had previously recognized three distinct species (within *Pleomele*) based on morphological differences: *P. hawaiiensis* (*sensu stricto*), *P. kaupulehuensis* St. John, and *P. konaensis* St. John. These three species were distinguished by leaf width and the perianth tube length. The perianth of *P. hawaiiensis* is 37–40 mm long with a perianth tube longer than 26 mm whereas the perianth of *P. konaensis* is less than 37 mm, with a perianth tube less than 23 mm, and the perianth of *P. kaupulehuensis* is greater than 43 mm long. The leaf width of *P. hawaiiensis* and *P. konaensis* is less than 22 mm compared to the leaf width of *P. kaupulehuensis* being greater than 23 mm [19]. The most recent treatment of these species combined them within the single species *P. hawaiiensis* (*sensu lato*) [17]. As such, it is also important to examine their population differentiation and genetic variation to gain a better understanding of their interrelations. 

There were two objectives of this study. First, to investigate the genetic structure within and among populations of the endangered species *C. hawaiiensis*. In doing so, comparisons will also be made of the level of diversity within populations of different size. Understanding the population genetic structure of the endemic Hawaiian *Chrysodracon* species will be desirable to provide the insight needed for proper conservation strategies to preserve the biodiversity of island ecosystems, reveal the evolutionary stages of those species, and address genetic resource problems that those populations are facing. It will also provide appropriate recovery suggestions for collecting the seeds and artificial pollination from those populations to incorporate the maximum genetic variation in these efforts. To best examine the population structure of an endangered species, it is also necessary to analyze the population structure of a non-endangered congener species for comparison. Therefore, our second objective was to conduct a genetic survey of *Chrysodracon auwahiensis* (Lu & Morden), a non-endangered species endemic to Maui (Figure 1) and Molokai Islands, to estimate the level and distribution of genetic diversity among populations. There are several extant populations of *C. auwahiensis* with thousands of individuals present. After completing the population genetics study of both *C. hawaiiensis* and *C. auwahiensis*, a comparison between them will provide an understanding of the type of variation that possibly was present in populations of *C. hawaiiensis* prior to habitat degradation and alteration. Knowledge of the population structure and level of variation will assist in formulating management practices for this species.

## 2. Results

In general, the genetic diversity measures in both random amplified polymorphic DNA (RAPD) and inter simple sequence repeat (ISSR) analyses were very similar and results obtained were highly compatible. Overall, the genetic diversity values were lower in RAPD than ISSR analyses and the values for population differentiation were higher in ISSR than RAPD analyses.

### 2.1. Random Amplified Polymorphic DNA (RAPD) and Inter Simple Sequence Repeat (ISSR) Profiles

Of the 80 RAPD primers and 48 ISSR primers screened, 11 and three (respectively) produced repeatable amplification in *C. hawaiiensis* that were scored for band presence/absence. These yielded 180 RAPD and 49 ISSR markers (229 total) scored (Table 1). Combined, there were 218 markers (95%) that were polymorphic. Population specific diagnostic RAPD and ISSR markers were present (although polymorphic) in each of the populations. Three such markers were found among Kohala, Manuka and Puu Waawaa, plants, and four among Kaupulehu plants.

The same 11 RAPD and three ISSR primers were also used to produce repeatable amplification products in *C. auwahiensis* (Table 1). A total of 198 RAPD and 40 ISSR markers (238 total) were scored and 235 markers (98%) were polymorphic. Each of the five populations also had population specific diagnostic markers (also polymorphic). Auwahi, Iao Valley, Kanaio, and Kauaula each had two diagnostic markers, whereas Makawao had eight.

Levels of RAPD and ISSR variation in *C. hawaiiensis*, measured by the percentage of polymorphic markers, exhibited slight differences among populations and displayed a similar relationship to the number of individuals sampled in each population (Table 2). The Puu Waawaa population was the largest population (200 individuals and 20 sampled) and was the most variable (68% and 73% polymorphism for RAPD and ISSR, respectively). The other three populations were considerably smaller (20–50 individuals) and had approximately the same sample size (10–13 individuals), and had similar values of polymorphism.

The amount of RAPD and ISSR variation found in populations of *C. auwahiensis* was relatively consistent among the five populations sampled, ranging from 70%–86% and 60%–73% for RAPD and ISSR, respectively (Table 2). All populations demonstrated greater levels of polymorphism than any *C. hawaiiensis* population. The two largest populations, Auwahi and Kanaio, also displayed the greatest level of polymorphism among individuals although the differences do not appear great and may be consistent with the number of plants sampled rather than population size. The two West Maui populations, Iao Valley and Kauaula, with the smallest estimated population size, show a reduced level of polymorphism among markers. Makawao, with approximately the same estimated population size as the West Maui populations, demonstrated only slightly higher polymorphism than the West Maui populations.

Values of estimated heterozygosity (*H*) similarly reflected sample sizes in *C. hawaiiensis* for both RAPD and ISSR data (Table 2). Interestingly, this pattern is not the same when examining *H* with only polymorphic markers. Kohala plants displayed higher values than was found among Puu Waawaa plants suggesting the Kohala population may have fewer polymorphic markers, but higher expected heterozygosity among those that are polymorphic. Heterozygosity estimates using all markers showed a pattern different from those of the polymorphic markers with much lower variation (Table 2). Total mean estimated heterozygosity over all markers in *C. hawaiiensis* was 0.254 (RAPD) and 0.316 (ISSR).

Estimated *H* in *C. auwahiensis* did not show great differences among populations (Table 2) but, rather, values were relatively consistent even among those with the smallest sample size (Iao Valley and Kauaula). The highest *H* value was found among the Makawao plants for the RAPD markers. However, values for ISSR markers did more consistently reflect the population size with both Auwahi and Kanaio, both higher than other populations, especially for *H* values based on polymorphic markers alone. The total mean estimated heterozygosity over all markers in *C. auwahiensis* was 0.401 (RAPD) and 0.352 (ISSR).

Shannon’s Diversity Index (SDI) estimates based on RAPD analysis demonstrated very little difference among populations within each species, but was consistently lower among populations of *C. hawaiiensis* compared to *C. auwahiensis* (Table 2). Values among populations of *C. hawaiiensis* ranged from 1.552–1.588 (1.576 among all individuals), whereas values of *C. auwahiensis* ranged from 1.675–1.693 (1.696 among all individuals). SDI from ISSR data similarly showed little variation among populations, but in contrast to the RAPD data, there was nearly a complete overlap of values between the two species (1.333 to 1.440 and 1.377 to 1.413 for *C. hawaiiensis* and *C. auwahiensis*, respectively).

### 2.2. Population Genetic Structure

Analysis of molecular variance population analyses of *C. hawaiiensis* indicate that variation within, and among, populations are similar based on combined RAPD and ISSR markers, with slightly more variation accounting for among populations (54%) than within populations (46%) (Table 3). The Φ_ST_ value of 0.536 (*p* = 0.001) suggests there is significant differentiation among the populations. In contrast, analysis of AMOVA among *C. auwahiensis* populations indicates there is considerably higher variation within populations (65%) than among them (35%). The Φ_ST_ value is also lower, 0.355 (*p* = 0.001), suggesting less differentiation among populations within this species.

### 2.3. Genetic Similarity Indices

Patterns of genetic similarity, as measured by RAPD and ISSR markers, were very consistent among populations for both species. Although some patterns were more clearly resolved with RAPD data as compared to the ISSR data and could be interpreted as the RAPD data being a more sensitive measure, this is likely an artifact of more genetic markers being measured for RAPD (180) than ISSR (49) analyses. As such, combined data analyses will be presented here; data for independent analysis is available from the authors.

Genetic similarity within and among populations was calculated using the Gower similarity coefficient analysis [20,21], and clearly reflect their relationships within each species (Table 4). As expected, individuals were most similar to members in their own population for both species. Intrapopulation similarity was relatively consistent among all populations for both *C. hawaiiensis* and *C. auwahiensis* with values ranging from 75%–87%; the lone exception to this was the Manuka population with only 66% similarity among individuals. Interpopulation comparisons within *C. hawaiiensis* were relatively consistent with values ranging from 47% (Manuka and Kaupulehu) to 60% (Puu Waawaa and Kohala). However, there was no clear indication of any clustering among population. In contrast, comparisons of the five *C. auwahiensis* populations examined suggest three population clusters. Plants from Iao Valley and Kauaula shared the highest similarity (67%) and plants from Auwahi and Kanaio shared an equally high value (66%); similarity among these two groups to populations of the other or to the Makawao population all ranged from 51%–55%, much lower than the values within each population cluster.

**Figure 2:**
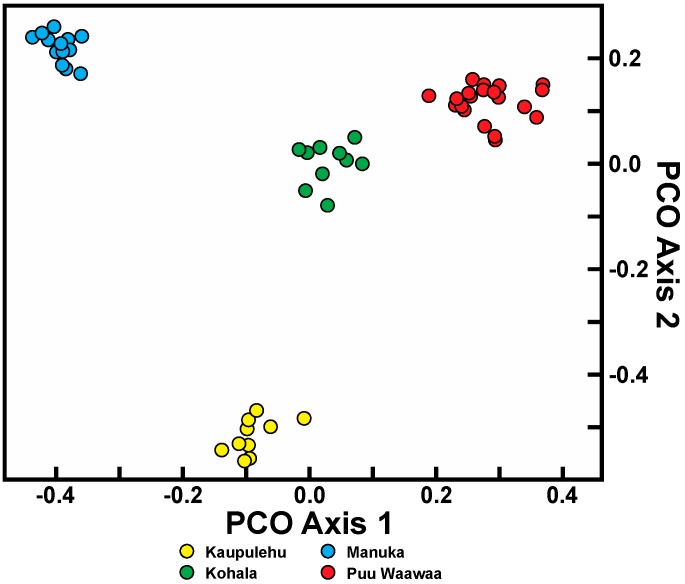
Principal coordinate analysis of combined random amplified polymorphic DNA and inter simple sequence repeats dataset using all scored markers for *Chrysodracon hawaiiensis*. PCO axis 1 and 2 accounted for 18.8% and 18.2% of the variation, respectively.

**Figure 3:**
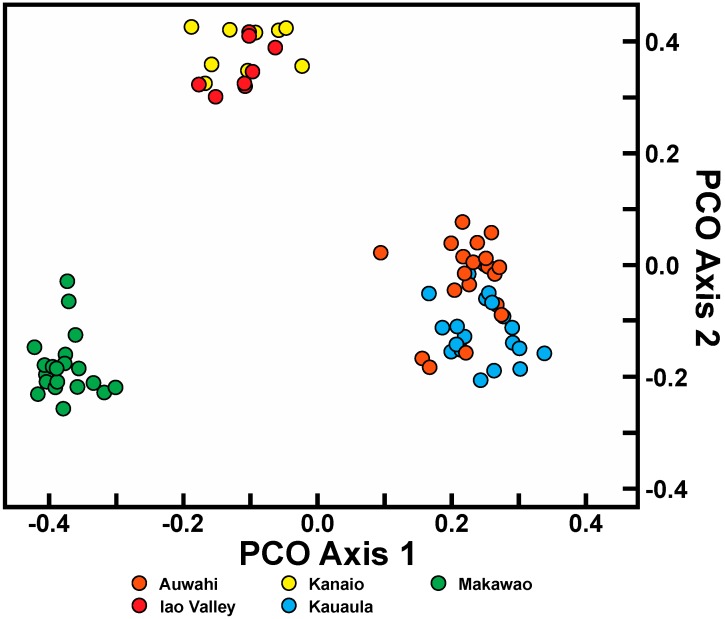
Principal coordinate analysis of combined RAPD and ISSR dataset using all scored markers for *Chrysodracon auwahiensis*. PCO axis 1 and 2 accounted for 17.1% and 11.2% of the variation, respectively.

Principal coordinate analysis (PCO) of the combined datasets for each species was consistent with the relationships based on genetic similarity and AMOVA. For *C. hawaiiensis*, the four populations examined were clearly distinct from one another (Figure 2). The first axis distinguishes the southernmost population of Manuka from northern populations of Puu Waawaa, Kohala, and Kaupulehu. The second axis distinguishes the Kaupulehu population from the other three along axis 2. In contrast, PCO analysis of the *C. auwahiensis* populations demonstrated three clusters, as suggested by similarity values (Figure 3). The Auwahi and Kanaio populations clustered together (with some degree of structure evident), and the West Maui populations of Iao Valley and Kauaula clustered together. Only the Makawao population was completely distinguishable from other populations.

**Figure 4:**
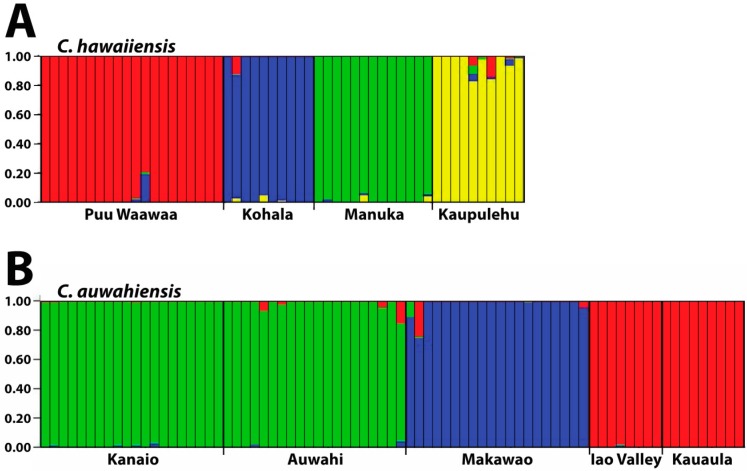
Genetic STRUCTURE bar graph of *Chrysodracon* species from combined RAPD and ISSR data. Individuals genotyped in this study are represented by a single vertical bar partitioned into colored segments that represent the individual’s probability of belonging to a particular group (K). (**A**) *C. hawaiiensis*, 53 individuals, K = 4; and (**B**) *C. auwahiensis*, 77 individuals, K = 3. Graphs represent one of 20 iterations from the indicated K value for each species.

Results from the STRUCTURE analysis of the combined RAPD and ISSR data are consistent with the results of the similarity and PCO analyses (Figure 4). For *C. hawaiiensis* (Figure 4A), the four populations sampled formed four distinctive groups (K = 4). Within populations, there was a slight indication of mixing of genotypes among some individuals. For *C. auwahiensis* (Figure 4B), the five populations sampled formed only three groups (K = 3) indicating some of the populations are not distinct. The Auwahi and Kanaio populations and the Iao Valley and Kauaula populations were genetically indistinguishable. As with *C. hawaiiensis*, there was a slight indication of mixing of genotypes in some individuals.

## 3. Discussion

### 3.1. Relative Genetic Variation

The present study is the first DNA-level examination within and among populations of *Chrysodracon* species, and establishes a baseline by which comparisons with other species (*Chrysodracon* or other dracaenoids) may be made. Genetic diversity within both of the species was moderate compared to other Hawaiian taxa examined, while among population differentiation was very significant. Percent polymorphism at the species level was 92% for all individuals of *C. hawaiiensis*, and this is higher than what has been found in many other taxa (*Dubautia ciliolata*: 70% and *Dubautia scabra*: 59% [22]; *Touchardia latifolia*: 51% [9]; *Alphitonia ponderosa*: 47%; and *Colubrina oppositifolia*: 41%, [10]). However, the population level variation was lower and in a moderate range compared to most other Hawaiian species [10,22]. Notably, the endangered species *C. hawaiiensis* shows similar genetic dynamics, as did the common species *C. auwahiensis*. Since these are long-lived plants, both species still maintain considerable genetic variation reflective of what may have existed prior to the start of the species decline.

Levels of genetic variation based on percent polymorphism indicate that *C. hawaiiensis* (70%) exhibited moderate levels of relative genetic diversity in comparison to *C. auwahiensis* (90%). Populations within *C. hawaiiensis* showed lower diversity (ranging from 50%–68%) than for the species as a whole. Similarly, levels of variation within populations of *C. hawaiiensis* show a similar trend. Populations within *C. auwahiensis* also showed lower diversity (ranging from 70%–86%) than for the species as a whole. Similarly, levels of variation within populations of *C. auwahiensis* also show a similar trend.

Both species are long-living woody perennial tree plants. Unfortunately, natural regeneration of young seedling in the field for either species was not evident. There were no reported wildfires destroying the forest, at least after 1947 (Hank Oppenheimer, Maui PEP Coordinator, personal communication) [23]. As such, the extant genetic diversity is likely representative of the diversity present for at least the past 100 years for *C. auwahiensis*, and the same is likely true for *C. hawaiiensis*. Most extant plants are old mature trees in populations that have probably experienced minimal impact from genetic drift despite declining population size. The endangered species *C. hawaiiensis* has similar, although slightly lower, estimated total polymorphism, heterozygosity, and Shannon’s diversity index over all polymorphic markers as compared to the more common *C. auwahiensis*.

### 3.2. Population Size and Genetic Diversity

Genetic diversity within populations reflected the estimated population size in species by both RAPD and ISSR analyses. Estimated heterozygosity over all loci and estimated genetic diversity was higher in the common species *C. auwahiensis* than in the endangered species *C. hawaiiensis*. These data suggest that *C. hawaiiensis* populations were, at one time, much larger, and the reduction in population sizes have been recent with some loss in genetic variation. In *C. hawaiiensis*, the estimated population size of Puu Waawaa was the largest and had higher genetic diversity. The other three populations have similar, but markedly lower, genetic diversity. The disparity in the levels of diversity is undoubtedly related to the estimated population sizes. Individuals in those populations are all long-lived old mature plants, and no evidence of seedlings or juveniles in the wild have been recorded (personal observation; Nick Agorastos, Hawaii NARS staff, personal communication). Both species have frequently produced flowers and seeds, but the lack of seedlings found during several years of observation is likely because of invasive weeds and insects (personal observation; H. Oppenheimer, Maui PEP, and Nick Agorastos, Hawaii NARS Coodinator, personal communication). Attrition of individuals from populations without subsequent regeneration may have contributed to the levels of variation now seen there.

Trends in population variation for *C. auwahiensis* were as predicted. The southern East Maui populations of Auwahi and Kanaio are larger and distributed over a wider geographical area compared to the Makawao or West Maui populations of the Iao Valley and Kauaula, and also showed the greatest genetic diversity. The Makawao population is distinct from the populations of East Maui or West Maui in genetic similarity analysis, and this population’s habitat is a mesic to wet forest rather than the dry forests of the other populations. Overall, data for *C. auwahiensis* indicates that populations encompassing a larger geographical area retain higher genetic diversity, but not greatly so, compared to those encompassing smaller or more isolated areas. The diversity within populations of *C. auwahiensis* is more similar than the greater ranges in diversity within populations of *C. hawaiiensis*, and is likely a consequence of all Maui populations still being relatively larger in size.

### 3.3. Distribution of Variation

The majority of variation in *Chrysodracon hawaiiensis* was found within, rather than among, populations although this difference was not great. In contrast, variation in *C. auwahiensis* populations was much greater within populations than among populations. It has been shown that long-lived plants, especially trees such as these, typically harbor a greater percentage of their variation within populations [24,25]. The study here supports these conclusions for *C. hawaiiensis*, but not *C. auwahiensis*. This may be a reflection of *C. auwahiensis* having two sets of populations that were genetically indistinguishable, although the three population clusters were very distinct. The numbers of diagnostic alleles unique to each population are possibly signs of differentiation among the populations following selection or genetic drift from the ancestral genetic environment. On the other hand, these alleles may be the representative of new mutations (such as deletions or insertions) that have appeared within populations following their initial dispersal after speciation events.

### 3.4. Population Differentiation

Isolation, created by geographical distance and subsequent fragmentation, has provided the initial means for divergence in both species. Since there is limited (if any) gene flow in both species due to habitat alteration between the populations, this divergence is likely to continue. Considerable population differentiation occurs in the four populations within *C. hawaiiensis*. As should be expected, the most geographically isolated of the populations, Manuka, on southern Hawaii Island, is genetically distinct from the other three populations on northern Hawaii Island. Unexpected was the low similarity of the Kaupulehu population from the others although, geographically, in close proximity. Interpopulation similarity of Kaupulehu is the lowest of any population comparisons, including the geographically-close Puu Waawaa population, suggesting this population is uniquely distinct from the others. The habitat at Kaupulehu is also distinct in that plants occur in wet, deep valleys on Hualalai Volcano, rather than the more exposed and/or drier habitats associated with the other populations.

For *C. auwahiensis*, analyses indicate there are three genetic population clusters among the five geographic locations examined. On West Maui (Iao Valley and Kauaula populations) and on southern East Maui, close relationship were anticipated because of their close geographic proximity and similarity of habitats. The habitat for populations on West Maui are typically wet soils in deep valleys. On southern East Maui, the habitat is very dry and exposed. The most genetically distinctive population on Maui is the northern East Maui Makawao population where within population similarity is the highest and interpopulation similarity the lowest. This distinctive population has a typically very wet habitat.

Reproductive biology in these species has not been examined up to date, but anecdotal evidence in the course of conservation work suggests that pollen and seed movement among populations is related to bird activities [26]. Neither *C. hawaiiensis* nor *C. auwahiensis* has had seedlings observed since the last century mainly due to introduced animals eating the leaves and young shoots, and the numbers of introduced animals drastically increasing in the forests in recent years [15]. *Chrysodracon* species have large bell-shaped yellowish flowers producing dark berry fruit, and have been hypothesized to share an association with birds for pollination and seed dispersal [26]. Although several potential factors may be important in limiting gene flow at this site (i.e., pollinating and seed-dispersing birds are now extinct), the separation of these populations is likely due to human habitat destruction and invasive species, such as pigs, goats, cattle, deer, rats, slugs, and alien plants. There are several lines of evidence to suspect continued differentiation among populations: (1) individuals among populations of both species share low genetic similarity; (2) gene flow among populations is restricted; and (3) localized inbreeding (or, in the extreme, self-fertilization) may be occurring due to lack of pollinators and Allee effects [27] within populations, which will result in a reduction of variation within populations. Dry forests are typically associated with leeward coast regions of all islands. *Chrysodracon* species typically survive on steep hillsides or lava substrates with well-drained soils. Thus, seed dispersal and gene flow among island populations may have been considerably greater prior to Polynesian inhabitation and the large-scale destruction of low elevation forests [28], extinction of bird species that followed, invasive weeds competition, and animal and slug grazing pressure [15].

## 4. Materials and Methods

### 4.1. Plant Collection and DNA Extraction

Leaf tissues were randomly collected from plants in four extant populations of *C. hawaiiensis* on Hawaii Island and five extant populations of *C. auwahiensis* on Maui Island (Hawaii State endangered species permit No. P-159 for *C. hawaiiensis*; special use permits of natural area reserves system (NARS) for both species were also obtained from the Hawaii Division of Land and Natural Resources (DLNR) (available upon request)). The plants frequently grow on rocky and inaccessible cliffs and, in some cases, sampling was limited due to safety concerns. Since these are rare species, a voucher specimen representative of each population was identified in the collections at Bernice Pauahi Bishop BishopMuseum (BISH) and is listed in Table 5 along with estimates of population size, locality, voucher information, and number of individuals collected. Total genomic DNA was extracted from fresh leaf tissue using the CTAB (cetyltrimethylammonium bromide) extraction protocol [29] with modification [30], or from silica dried samples using the Qiagen DNeasy Plant Mini kit according to the manufacturer’s instructions (Qiagen Corporation, Valencia, CA, USA). Samples were accessioned into the Hawaiian Plant DNA Library [30,31].

### 4.2. Genetic Analysis

Approximately 25 ng of DNA was amplified via the polymerase chain reaction (PCR) performed in a MJ Research Thermal PCR machine (GMI, Inc., Ramsey, MI, USA) in 15 μL volume reactions. Conditions for RAPD reactions were 0.2 μM random 10-mer oligonucleotide primers, 0.2 mM each of dNTP, 1× Taq polymerase PCR buffer, 1.5 mM MgCl_2_, 0.01 g/mL concentration 1% Bovine Serum Albumins (BSA) in the total reaction volume, and 1 unit of *Taq* polymerase (Promega, Madison, WI, USA). RAPD PCR conditions were for one cycle at 94 °C for 3 min, 35 °C for 30 s, and 72 °C for 2 min, followed by 43 cycles at 94 °C for 45 s, 35 °C for 30 s, and 72 °C for 2 min, and a final cycle at 94 °C for 45 s, 35 °C for 30 s, and 72 °C for 6 min. Conditions for ISSR reactions were 0.4 μM primer, 0.2 mM each dNTP, 1× Taq polymerase PCR buffer, 2.5 mM MgCl_2_, 5% 0.01 g/mL concentration BSA in the total reaction volume, and 1 unit of Taq Polymerase (Promega, Madison, WI, USA). ISSR PCR conditions were 94 °C for 90 s, followed by 34 cycles of 94 °C for 40 s, 45 °C for 45 s, and 72 °C for 90 s, followed by 94 °C for 45 s, and 45 °C for 45 s, ending with 5 min at 72 °C after cycling was completed.

Amplification products were mixed with loading dye (20 mm EDTA, 10% glycerol, 1% sarcosyl with bromophenol blue and xylene cyanol) and separated in 1.5% agarose gels in 0.5× TBE (tris-borate-EDTA) buffer with 125 ng ethidium bromide per liter. Sizes of the amplification products were estimated by comparison to a Promega 100 bp ladder (Promega, Madison, WI, USA). RAPD primers (Operon Technology, Alameda, CA, USA; kits OPA-OPI) and ISSR primers (University of British Columbia Primer Kit #9, Vancouver, BC, Canada) were screened for amplification of *Chrysodracon* DNA, and selected primers were then used for amplification of all individuals. Selected ISSR primers were 5007 (ACACACACACACACAC-C), 5009 (ACACACACACACACAC-T), and 5028 (GAGAGAGAGAGAGAGA-YT). Molecular markers were identified by the primer used to generate them and the approximate size of the band as estimated from a 100 bp ladder.

The reproducibility of amplification was tested for each primer prior to data collection. GelAnalyzer 1D image analysis software (Dr. Istvan Lazar, www.gelanalyzer.com) was initially used to estimate the number of base pairs represented by each amplified fragment and manually adjusted based on eye observation. Loci were scored as diallelic (1 = band present, 0 = band absent). Gels were scored independently by the first and second authors to produce unbiased and unambiguous analysis of RAPD and ISSR amplifications.

### 4.3. Data Analysis

Assumptions related to RAPD marker analysis were described by Lynch and Milligan [32] and also apply to ISSR analysis. RAPD and ISSR markers were determined to be polymorphic if estimated allele frequency was less than 95%. In practice, a population marker was considered polymorphic when amplification was present in one or more individuals of the population or if a null (no amplification) occurred in one or more individuals. Absence of a marker within a population, although present in others, was assumed to indicate the individual to be a null/null homozygote rather than there having been a loss of the locus. Expected heterozygosity was calculated for each population (*H_S_*) and species (*H_T_*) for each locus as follows: *H* = 1 − (*p^2^* + *q^2^*) where *p* is the frequency of the amplified allele and *q* is the frequency of the null allele; allele frequencies were estimated from the number of null/null homozygotes present in the population [33]. Lynch and Milligan [32] point out that only markers present with an observed frequency of less than 1 − (3/*N*) (where *N* represents the sample size) are used to reduce a potential bias when analyzing dominant markers. Summary statistics of average similarity measures (means, standard errors, and *t*-tests) were calculated using Excel (Microsoft Office 2007, Microsoft District for Pacific Northwest, Bellevue, WA, USA). Distribution of genetic variation within and among populations was estimated using Shannon’s diversity index (*H*) [34]. Shannon’s diversity index (*H*) was calculated as:

*H_O_* = −Σ *p_i_*·log2 *p_i_*

where *p_i_* is the frequency of a given RAPD or ISSR phenotype within a population or species group.

Genetic structure among populations of each species was measured by four different methods. Analysis of molecular variance (AMOVA) [35] estimates population differentiation directly from molecular data and was implemented in GenAlex 6.1 [36]. The AMOVA approach computes Φ_ST_, a statistic analogous to *F_ST_*, that estimates the level of genetic differentiation between populations and ranges from 0 (complete genetic homogeneity) to 1 (complete genetic separation). Population-grouped similarity coefficients based on Gower general similarity coefficient [20,21] were used to calculate an average similarity value within and among populations. Similarity values range between 0 and 1, the former indicative of complete genetic dissociation and the latter genetic identity. Principal coordinate analysis (PCO) was used to graphically represent genetic relationships among each individual using MVSP 3.0 (Multi-Variate Statistical Package; Kovach Computing Services 1986–2011, Kovach Computing Services, Anglesey, Wales) based on Gower general similarity coefficient [20,21]. A Bayesian algorithm, as implemented in STRUCTURE version 2.3.4 [37,38], was used to define genetic groups within each species. This algorithm infers genetic discontinuities from individual multilocus genotypes without a priori knowledge of geographic location or taxonomy. The default settings of the program were used, including an admixture model. To determine the most likely number of groups (K) in the data, a series of analyses were performed from K = 1 to 7 or 8 (upper limit determined by the number of populations plus three [39]), using a burn-in period and MCMC (Markov Chain Monte Carlo) both set at 100,000 repetitions, with twenty iterations per K [40]. These results were examined using the ∆K method [37] to identify the most likely number of groups in the data using STRUCTURE HARVESTOR [41]. 

## 5. Conclusions and Conservation Implications

Results of this study demonstrate several important factors regarding the genetic diversity and structure within these species. Patterns of genetic diversity and genetic differentiation within and among populations are similar for both species examined. However, the level of variation found in *C. hawaiiensis*, an endangered species with smaller and more isolated populations, is consistently lower than that found in *C. auwahiensis*, a non-endangered species with much more extensive populations. Populations of *C. hawaiiensis* have been in decline for at least 50 years (Nick Agorastos, Hawaii NARS staff, personal communication), yet a level of genetic diversity nearly equal to that of a non-endangered congener occurring in similar habitats suggests that the effects of inbreeding within populations have not yet had a significantly deleterious impact on their vigor. Genetic diversity at the species level remains very high, as levels of polymorphism are above 90% and nearly equal to those species known to have the highest level of genetic diversity yet measured among Hawaiian species [11]. This is likely a reflection of the species habit (long-lived trees) and habitat (mostly dry forests) that promote slow growth in individuals. Since little to no recruitment of plants within populations has been observed, it is probable that the genetic diversity observed is from individuals that have survived in these environments since before the populations went into decline and that loss of variation is because of population attrition rather than loss of alleles through inbreeding. There are approximately 20 very endangered individuals of *C. hawaiiensis* in scattered locations at Hawaii Volcano National Park (HAVO) that were not examined here. Any future study of these species should include the HAVO population.

Long-term survival of *C. hawaiiensis* will not be possible by simply maintaining current population numbers without active conservation management. The impacts from animal grazing pressure have played a pivotal role in the erosion of plant diversity of Hawaiian dry forests. For *C. auwahiensis*, the additional pressure on the populations by invasive weeds competing with seedlings and invasive slugs that eat seedlings are further threats (Hank Oppenheimer Maui PEP, personal communication). The consequence has been zero seedling recruitment in these populations. For future conservation work, it has been suggested that seed collections be made from different populations of each species to increase the genetic variation and benefit the long term survival of endangered species. Based on the polymorphism data, *C. auwahiensis* on Maui still maintains enough genetic variation (70%–86%) for each population, thus seed collection from individual populations should be made broadly.

Future research should focus on the reproductive biology of these species. Virtually nothing is known regarding the pollination, seed survival, and growth of these plants. Pollination observations and open flower vs. closed flower seed set experiments would provide the necessary information regarding inbreeding among the species. Seed germination experiments would be beneficial for understanding breaking of dormancy, germination rates, and seedling survival. Seedlings would then also be available for potential population reintroduction.

Several conservation measures are recommended to protect both species. First, and most importantly, any threats to the plants at the early stages of their development must be removed. This can only be accomplished by building predator-proof fences that can exclude introduced herbivorous animals, particularly goats, from those areas. Some snail baits have recently been approved for use in conservation areas, and strategies for their use should be developed to implement this control where snails and slugs are a factor. Second, mature plants readily flower and fruit, and efforts should focus on establishing an ex situ seed bank for both species. Care should be made while collecting to target widely-spaced plants to capture the maximum genetic diversity possible [42]. Third, growing plants ex situ for future reintroduction into the source populations when they have attained a size sufficient to withstand existing threats (i.e., slugs and goats) would help maintain the population’s integrity until other measures have been implemented that will allow natural recruitment. Fourth, because individual population variation of *C. hawaiiensis* is in decline, yet total species variation is high, limited mixing of population progeny is recommended to maintain higher levels of genetic diversity that has been shown to be beneficial for the long-term survival in a wide variety of species [43]. The loss of genetic variation has been shown to have harmful effects on fitness of individuals of populations [33,44]. Possible problems associated with outbreeding depression that could occur from mixing different population progeny are minimal, if present at all, and are far less than potential future problems associated with inbreeding depression. Performing hand-pollination crosses among plants from different populations and growing the individuals from such crosses with the purpose to outplant them might also attain this. 

## Figures and Tables

**Table 1:** Random amplified polymorphic DNA (OPA-OPD) and inter simple sequence repeat primers examined and the number of genetic markers scored. Primer name, number of total scored amplification products (*N*), number of polymorphic bands (*P*), percent polymorphism (%*P*), and the range of size (in base pairs) for amplified products (size) for *Chrysodracon auwahiensis* and *Chrysodracon hawaiiensis*. Subtotal for RAPD and ISSR primers separately; and total for RAPD and ISSR primers combined.

Primer	*N*	*P*	%*P*	Size	*N*	*P*	%*P*	Size
	*Chrysodracon hawaiiensis*				*Chrysodracon auwahiensis*			
OPA-02	14	13	93	300–1600	21	20	95	175–1600
OPA-09	13	13	100	300–1600	18	18	100	350–2300
OPB-04	19	18	95	260–1500	12	12	100	300–1800
OPB-07	23	23	100	260–1500	19	19	100	170–2400
OPB-08	20	20	100	150–1300	20	20	100	200–1500
OPB-14	19	19	100	150–1600	20	20	100	250–1500
OPC-07	10	9	90	200–2000	14	14	100	180–2000
OPD-02	16	14	88	200–1000	16	16	100	280–1500
OPD-05	16	15	94	200–1200	21	21	100	170–500
OPD-12	11	11	100	350–1000	14	14	100	180–1800
OPD-15	19	18	95	300–2000	23	21	91	300–2000
Subtotal	180	173	96	-	198	195	98	-
ISSR-5007	22	21	95	250–1800	15	13	87	300–1800
ISSR-5009	13	12	92	480–1500	12	9	75	350–1300
ISSR-5028	14	12	86	300–1600	13	13	100	300–1800
Subtotal	49	45	92	-	40	35	88	-
TOTAL	229	218	95	-	238	230	97	-

**Table 2:** Genetic variability among populations of *Chrysodracon hawaiiensis* (*C. hawaiiensis*) and *Chrysodracon auwahiensis* (*C. auwahiensis*) based on RAPD and ISSR analyses. Percentage of polymorphic markers (%*P*), estimated mean heterozygosity over all markers (*H*), estimated mean heterozygosity over polymorphic markers (*H* (*P*)), and Shannon’s Diversity Index (SDI). The slash (/) separates values for RAPD and ISSR analyses, respectively.

Population	%*P*	*H*	*H* (P)	SDI
*Chrysodracon hawaiiensis*				
Kaupulehu	54/57	0.168/0.210	0.314/0.352	1.552/1.395
Kohala	53/45	0.184/0.187	0.350/0.416	1.588/1.333
Manuka	50/65	0.152/0.223	0.301/0.340	1.576/1.410
Puu Waawaa	68/73	0.218/0.276	0.322/0.375	1.582/1.440
All individuals	96/92	0.254/0.316	0.363/0.418	1.576/1.450
*Chrysodracon auwahiensis*				
Auwahi	85/70	0.241/0.216	0.302/0.309	1.675/1.407
Iao Valley	77/60	0.238/0.184	0.309/0.226	1.668/1.386
Kanaio	86/73	0.240/0.257	0.302/0.355	1.680/1.413
Kauaula	70/3	0.226/0.181	0.317/0.271	1.685/1.377
Makawao	80/65	0.263/0.217	0.363/0.230	1.693/1.394
All individuals	98/88	0.401/0.352	0.432/0.403	1.696/1.397

**Table 3:** Analysis of molecular variance (AMOVA) for 53 individuals in four populations of *C. hawaiiensis* and 77 individuals in five populations of *C. auwahiensis* based on combined RAPD, ISSR, and combined dataset analyses. Abbreviations: d.f., degrees of freedom; SSD, sum of squared deviation; MSD, mean squared deviation; Var. Comp., variance component; %T, percentage of total variance contributed by each component; *p*, probability of obtaining a more extreme component by chance alone; and Φ_ST_, the degree of differentiation between population divisions.

Source of Variation	Analysis	d.f.	SSD	MSD	Var. Comp.	%T	*p*	Φ_ST_
*C. hawaiiensis*								
Among Pops	RAPD	3	827.1	275.7	19.6	44	0.001	0.442
	ISSR	3	199.0	66.4	4.8	52	0.001	0.519
	Combined	3	1218.0	406.0	29.5	54	0.001	0.536
Within Pops	RAPD	49	1208.4	26.7	24.7	56	0.001	-
	ISSR	49	218.7	4.5	4.5	48	0.001	-
	Combined	49	1255.6	25.6	25.5	46	0.001	-
*C. auwahiensis*								
Among Pops	RAPD	4	975.0	243.7	14.5	35	0.001	0.347
	ISSR	4	192.7	48.2	2.9	40	0.001	0.401
	Combined	4	1167.7	291.9	17.5	35	0.001	0.355
Within Pops	RAPD	72	1973.2	243.7	14.5	65	0.001	-
	ISSR	72	316.7	4.4	4.4	60	0.001	-
	Combined	77	2298.9	31.8	31.8	65	0.001	-

**Table 4:** Matrix of the average of coefficient genetic percent similarity (based on Gower similarity coefficients [20,21]) within and among populations of *C. hawaiiensis* and *C. auwahiensis* from combined RAPD and ISSR dataset analysis.

Population No.	*C. auwahiensis*	1	2	3	4	5	Population No.	*C. hawaiiensis*	6	7	8	9
1	Auwahi	83					6	Kaupulehu	85			
2	Iao Valley	55	75				7	Kohala	54	85		
3	Kauaula	55	67	76			8	Manuka	47	50	66	
4	Kanaio	66	53	53	84		9	Puu Waawaa	54	60	56	87
5	Makawao	51	52	51	51	86						

**Table 5:** Samples analyzed for population genetic variation of *C. hawaiiensis* (Hawaii Island) and *C. auwahiensis* (Maui Island). Species classification, locality, estimated population size (*N*), number of plant per population sampled (*N*_s_), DNA accession in the Hawaiian Plant DNA Library (HPDL), and representative population voucher. The topographic map of for the populations’ locations of two species are shown on Figure 1. Due to species rarity, voucher specimens were not collected with this study, but representative specimens from each locality were deposited at the B. P. Bishop Museum (BISH) are indicated.

Species Locality	*N* ^1^	*N*_S_	HPDL	Voucher
*C. hawaiiensis*				
Kaupulehu	50	10	8170–8179	*J. D. Jacobi 251*
Kohala	20	10	8193–8202	*C. Christensen 1*
Manuka	50	13	8180–8182	*H. St. John 11343*
Puu Waawaa	200	20	8170–8179	*Y. Kondo 44*
*C. auwahiensis*				
Auwahi	600	20	6632–6644, 6661–6667	*H. St. John 26869*
Iao Valley	300	8	6591–6598	*J. C. Price 19*
Kanaio	600	20	6611–6630	*R.W. Hobdy 2552*
Kauaula	300	9	6599–6606	*D. R. Wood 11943*
Makawao	300	20	6607–6610, 6645–6660	*H. L. Oppenheimer H50221*

^1^ Estimated population size of *C. hawaiiensis* from N. Agorastos (Hawaii Natural Area Reserves System) and *C. auwahiensis* from H. L. Oppenheimer (Maui Nui Plant Extinction Prevention Program) (personal communication).
